# Appetite‐stimulating effects of once‐daily omeprazole in cats with chronic kidney disease: Double‐blind, placebo‐controlled, randomized, crossover trial

**DOI:** 10.1111/jvim.16268

**Published:** 2021-09-30

**Authors:** Ashley Spencer, Jessica M. Quimby, Josh M. Price, Sally MacLane, Shanna Hillsman, Patty Secoura, Jörg M. Steiner, M. Katherine Tolbert

**Affiliations:** ^1^ North Carolina State University, College of Veterinary Medicine Raleigh North Carolina USA; ^2^ The Ohio State University, College of Veterinary Medicine Columbus Ohio USA; ^3^ Department of Small Animal Clinical Sciences University of Tennessee, College of Veterinary Medicine Knoxville Tennessee USA; ^4^ Appalachian Animal Hospital Piney Flats Tennessee USA; ^5^ Gastrointestinal Laboratory, Department of Small Animal Clinical Sciences College of Veterinary Medicine and Biomedical Sciences, Texas A&M University College Station Texas USA

**Keywords:** activity, dysrexia, proton pump inhibitor, vomiting

## Abstract

**Background:**

Cats with moderate to advanced chronic kidney disease (CKD) often display clinical signs such as vomiting and decreased appetite, and frequently receive omeprazole or other acid suppressants despite a lack of evidence to support their use.

**Hypothesis/Objectives:**

To evaluate the effect of once‐daily PO omeprazole on appetite in cats with CKD. We hypothesized that omeprazole would improve subjective appetite assessments in cats with CKD.

**Animals:**

Fourteen client‐owned cats with International Renal Interest Society (IRIS) stage 2 or 3 CKD and hyporexia.

**Methods:**

Cats were prospectively enrolled in a multi‐institutional, double‐blinded, randomized, crossover study to evaluate the effect of a 14‐day trial of once‐daily PO omeprazole (1 mg/kg) or placebo (lactose gel capsule) on vomiting frequency and appetite. A daily log was completed by the owner during all treatment and rest periods to assess appetite using a subjective, qualitative, and 5‐point scoring system. Mixed model analyses of variance were performed to determine if average daily percentage food consumed or appetite score, as measured by subjective owner assessment, differed between treatments.

**Results:**

Compared to placebo, a negligible but statistically significant difference in percentage of food consumed was observed between treatments (*P* = .04) with once‐daily omeprazole treatment resulting in a 2.7% increase in food consumption compared to placebo. No significant difference, however, was found in appetite score, body weight, or serum creatinine concentration between treatments.

**Conclusions and Clinical Importance:**

Once‐daily omeprazole does not markedly increase appetite in cats with CKD and should not be used as a first‐line treatment in the absence of evidence of gastrointestinal ulceration.

**Abbreviations**
ANOVA: analysis of variance
BUN: blood urea nitrogen
CKD: chronic kidney disease
GI: gastrointestinal
IRIS: International Renal Interest Society
PPI: proton pump inhibitor

## 1 INTRODUCTION

Chronic kidney disease (CKD) is common and leads to substantial morbidity and mortality in older cats.^1^, ^2^, ^3^ The cause of CKD in cats is unknown and therefore the goals of treatment are to slow the progression of disease and improve quality of life. Cats with CKD often display clinical signs such as decreased appetite and vomiting, and current treatment recommendations include measures to address these signs.^4^ Empirical treatment of cats with CKD‐associated dysrexia or vomiting using a gastric acid suppressant, such as proton pump inhibitors (PPIs; eg, omeprazole), is common.^5^ However, recent epidemiological studies in humans have demonstrated an association of chronic PPI use and several adverse effects, including *Clostridioides difficile*‐associated diarrhea, dementia, pneumonia, micronutrient deficiencies, bone mineral density disorders, and renal disease.^6^, ^7^, ^8^ Given the common use and recent concerns for adverse effects of chronic PPI usage, the American Gastroenterological Association released guidelines and best practice advice for the indications and use of PPIs.^7^ One such indication in people is the use of PPIs for the treatment of end‐stage renal disease, a disease in which peptic ulceration can be observed.^9^, ^10^, ^11^, ^12^ Cats with CKD have been shown to have gastric mineralization and fibrosis, but not the ulcerative lesions frequently observed in people with CKD.^13^ Moreover, in a recent study, we were unable to detect a significant difference in gastric pH in cats with International Renal Interest Society (IRIS) stage 2 to 4 CKD compared to healthy, aged‐matched control cats.^14^ However, veterinarians and owners of cats with CKD often describe a positive effect of acid suppressant treatment on CKD‐related gastrointestinal (GI) signs, including improved appetite and decreased vomiting. Thus, it is possible that acid suppressants provide beneficial effects in cats with CKD that are independent of their effects on gastric pH.

To our knowledge, no studies have been performed to determine if omeprazole administration improves appetite or decreases vomiting in advanced‐stage CKD in cats. Accordingly, our primary objective was to qualitatively evaluate the effects of once‐daily PO omeprazole administration on subjective appetite assessments in cats with IRIS stage 2 and 3 CKD. Based on practitioner experiences, we hypothesized that once‐daily omeprazole would improve subjective appetite assessments in cats with CKD.

## 2 MATERIALS AND METHODS

### 2.1 Animals

Client‐owned cats from several academic referral hospitals and a local small animal practice were screened for enrollment. The original intent was to enroll cats with stable IRIS stage 3 CKD (serum creatinine concentration, 2.9‐5.0 mg/dL). However, recruitment of cats with advanced stages of CKD was challenging and modification to the enrollment criteria was made in the middle of the study to allow for inclusion of cats with IRIS stage 2 CKD (serum creatinine concentration, 1.6‐2.8 mg/dL). Inclusion criteria included a history of inappetence and other clinical signs thought to occur secondary to CKD (eg, vomiting). Before enrollment, cats were required to have a record of compatible clinical history, physical examination findings, and diagnostic evidence supportive of stable IRIS stage 2 or higher CKD. Cats receiving medications for treatment of sequelae related to CKD (eg, antihypertensive drugs, antiproteinuric drugs) were included if the medication had been initiated >2 weeks before study enrollment. Owners of cats receiving acid suppressants within 2 weeks of study entry were asked to discontinue the medication for a minimum of 7 days before enrollment. Exclusion criteria included a normal appetite, a history or suspicion of hepatic or pancreatic disease based on laboratory test results, a diagnosis of primary GI disease or diabetes mellitus, or the presence of destabilizing complications from underlying CKD, such as acutely progressive azotemia. Dietary management was not standardized, but the cat's diet (either type or amount offered) could not be changed for the duration of the study. Informed client consent was obtained from all owners using an institution‐specific informed client consent document.

### 2.2 Study design

**FIGURE 1:**
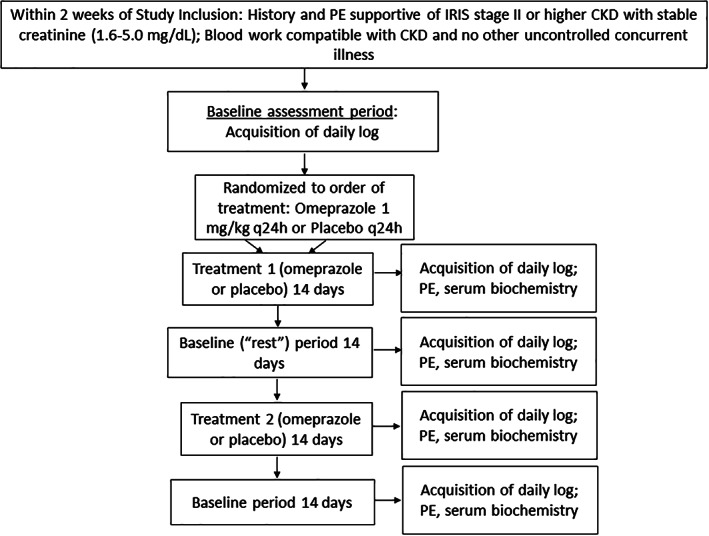
Flowchart describing assessment, enrollment, allocation, timeline, and experimental design for CKD cats treated with omeprazole in a double‐blinded, randomized, crossover study. Fourteen cats completed the study. CKD, chronic kidney disease

The study was a multi‐institutional, prospective, double‐blinded, randomized, crossover study comparing appetite in cats with ≥IRIS stage 2 CKD and a history of decreased appetite with or without vomiting after treatment with omeprazole or placebo. A timeline for the study is shown in Figure 1.

Before initiating the study, enrolled cats underwent a week of baseline assessment in which the owner completed a daily log (Figure S1). Owners continued to complete a daily log throughout the study period, including during all rest and treatment periods. Cats then were randomized into 2 groups where they initially received either omeprazole (omeprazole, 20 mg capsules; Lannett Company, Inc, Trevose, Pennsylvania) compounded into lactose‐filled gel capsules at a median dose of 1.1 mg/kg (range, 1.0‐1.2 mg/kg) given PO q24h or placebo (lactose‐filled gel capsule PO q24h) for 14 days. The goal of treatment was to achieve a dosage of approximately 1 mg/kg for omeprazole; thus, 1 enteric‐coated bead containing 1.1 mg omeprazole was given per kg body weight. The enteric‐coated beads were placed in lactose‐filled gel capsules such that capsules containing omeprazole beads and those containing placebo were indistinguishable from each other, either visually or by shaking the capsule. The investigators as well as cat owners were blinded to treatment order. Unblinded technicians (S. Hillsman, P. Secoura) placed appropriate labels on each container (ie, “Treatment A” or “Treatment B”) and the owners were given each bottle at the appropriate time for each treatment period. A preset order was determined for randomization and cats were assigned to treatment or placebo consecutively (AB or BA). Owners were instructed to administer medication 30 minutes before the morning meal. After both treatments, cats underwent a 14‐day rest period.

The following were performed at the beginning and end of all treatments and rest periods: physical examination (including measurement of body weight and blood pressure), serum biochemistry profile, estimation of body condition score (based on a 9‐point scale; Cat Body Condition Scoring System developed by The World Small Animal Veterinary Association [WSAVA], Dundas, Canada), and estimation of muscle condition score (Muscle Condition Scoring System developed by WSAVA) scored as normal or mildly, moderately, or severely impaired muscle condition. All laboratory tests were performed by the clinical pathology services at the respective enrolling institution. For each cat enrolled, physical examination and laboratory testing were performed by the same clinician and the same laboratory, respectively.

The daily log (Figure S1) consisted of questions related to medication compliance, appetite, activity, and the presence of nausea or vomiting. For the duration of study participation, a single person was instructed to fill out the daily log. Owners were instructed to document daily treatments administered as well as patient tolerance of the medication. The cat's daily appetite was assessed using both quantitative and qualitative measures. Appetite was qualitatively assessed by the owner as a subjective assessment of appetite. Appetite also was assessed quantitatively using a 5‐point scale as a percentage of food consumed related to the amount offered. The owner was instructed to characterize the amount of food consumed as a percentage of food offered to the cat on a given day (ie, 100% of food offered consumed, 75% of food offered consumed, 50% of food offered consumed, 25% of food offered consumed, or none food offered consumed). Second, the owner was instructed to express whether they subjectively felt the cat had a change in appetite compared to day 0 (1 day before treatment) and this assessment was expressed as decreased, unchanged, or increased. Voracity of appetite and change in activity also were assessed with instruction to the owner to note whether they observed increased, decreased, or unchanged food seeking behavior as well as rate of food consumption and activity level. The number of vomiting episodes also was recorded daily.

### 2.3 Statistical analysis

Sample size estimation based on a paired *t* test for mean difference in an AB/BA crossover design was performed before the study to determine the number of cats needed to detect a 20% increase in appetite score in the omeprazole treatment group compared to a placebo‐treated group. Using an α of .05 and a power of 0.8, 16 cats were identified as the target for enrollment.

Qualitative appetite and activity data were converted to clinical scores as follows: −1 = decreased appetite, 0 = unchanged appetite, or 1 = increased appetite or activity. Food seeking behavior also was scored: −1 = decreased, 0 = unchanged, or 1 = increased. These scores then were summed over each of the 14‐day treatment periods. The daily percentage of food consumed was averaged over each of the 14‐day treatment and rest periods. The total number of vomiting episodes was summed over each of the 14‐day treatment periods. The SAS statistical software package (SAS 9.4, Cary, North Carolina) was used for data analysis and Prism (Prism 9.0.0, GraphPad, La Jolla, California) was used to create plots. Mixed model analyses of variance (ANOVAs) were performed to evaluate percentage food consumed, qualitative appetite score, summed activity score, food‐seeking behavior, rate of food consumption, and sum of vomiting episodes for treatment differences. A binomial generalized estimating equation model was used to evaluate the odds of observing vomiting (yes/no) between treatments. Mixed model ANOVAs were performed to evaluate serum biochemical results related to renal function (creatinine, phosphorus, potassium, and blood urea nitrogen [BUN] concentrations) and body weight for differences between treatment and over time. The IRIS stages were incorporated in each model to account for CKD severity. The IRIS stage of 2 cats fluctuated between high stage 3 and low stage 4 between both treatment and rest periods during both treatments. For the purposes of analysis, these cats were classified as stage 3.

A paired *t* test was performed to assess for a placebo effect on appetite and activity scores, percentage of food consumed, and food‐seeking behavior. This evaluation was performed using IBM SPSS (SPSS 27, Armonk, New York).

A Shapiro‐Wilk test for normality and QQ normality plots were used to evaluate normality of ANOVA residuals. Levene's equality of variances test was used to evaluate equality of treatment variances. All statistical assumptions regarding normality and equality of variances were met. Statistical significance was defined as *P* ≤ .05.

## 3 RESULTS

After initial prescreening, where >100 cats were evaluated for study inclusion, 24 cats were screened in 1 of the participating hospitals for enrollment. Ten were excluded because of the presence of exclusion criteria identified during in‐clinic screening, including the presence of urinary tract infection, advanced cardiac disease, or severe unstable CKD. Sixteen cats (n = 4 Appalachian Animal Hospital, n = 3 University of Tennessee, n = 3 The Ohio State University, n = 6 North Carolina State University) met the inclusion criteria. One developed a urinary tract infection during the study and the owner of 1 cat withdrew the cat from the study before receiving both treatments. Fourteen of the 16 cats completed the study. Based on the clinical history obtained from the owner and available medical record review, all 14 cats had signs of inappetence as required for study inclusion. Four cats had vomiting and 7 cats had weight loss, poor muscle mass, or poor body condition. There were 9 spayed females and 5 castrated male cats; breeds included 7 domestic short hair, 4 domestic long hair, 2 Ragdoll, and 1 Maine Coon. Median age was 12.5 years (range, 6‐20 years). Median body weight at the time of study enrollment was 4.4 kg (range, 2.9‐11.0 kg). An equal number of cats (n = 7 each) was classified as having IRIS stage 2 and IRIS stage 3 CKD at study entry. Median body condition score was 5 (range, 3‐7 on a 9‐point scale). Four cats were described as having normal muscle condition scores, 6 as having mild muscle loss, and 4 as having moderate muscle loss. Serum total thyroxine concentration was measured in all 14 cats and was within the reference limits in all cats. Blood pressure measurements were acquired in all cats enrolled and were within the reference range. None of the cats included in the study had proteinuria. Eight cats were receiving a commercial therapeutic renal diet exclusively, whereas 4 were receiving a combination of a renal diet and other alternative sources of meat or alternative diets, and 2 cats were noted to not have tolerated a renal diet and were eating diets formulated for maintenance of adult cats. Treatments for the underlying renal disease as reported by the owners included SC fluids (7 cats), mirtazapine (4 cats), a probiotic (Fortiflora, Nestlé Purina PetCare Company, St. Louis, Missouri; 3 cats), amlodipine (2 cats), maropitant citrate (1 cat), omega‐3 fatty acids (2 cats), and darbepoetin (1 cat). Two cats had a history of constipation and were receiving a stool softener on a regular basis.

The study consisted of a combined total of 392 treatment days for all 14 cats receiving the 2 treatments, 14 treatment days on placebo and 14 treatment days on omeprazole. On 378 of those treatment days (96.4%), cats were reported to tolerate the respective treatment. In total, 12 doses of the placebo and 2 doses of omeprazole were missed. Missed treatments were caused by difficulty pilling, missed doses, or an episode of vomiting immediately after medication administration. In 8 cats, all doses for either treatment had been administered successfully. In particular, 2 owners had difficulty with medication administration leading to missed doses and also vomiting immediately after administration. Missed doses in these 2 cats accounted for 11 missed doses in total (10 of those during the placebo treatment period). The remaining missed doses were single episodes.

**FIGURE 2:**
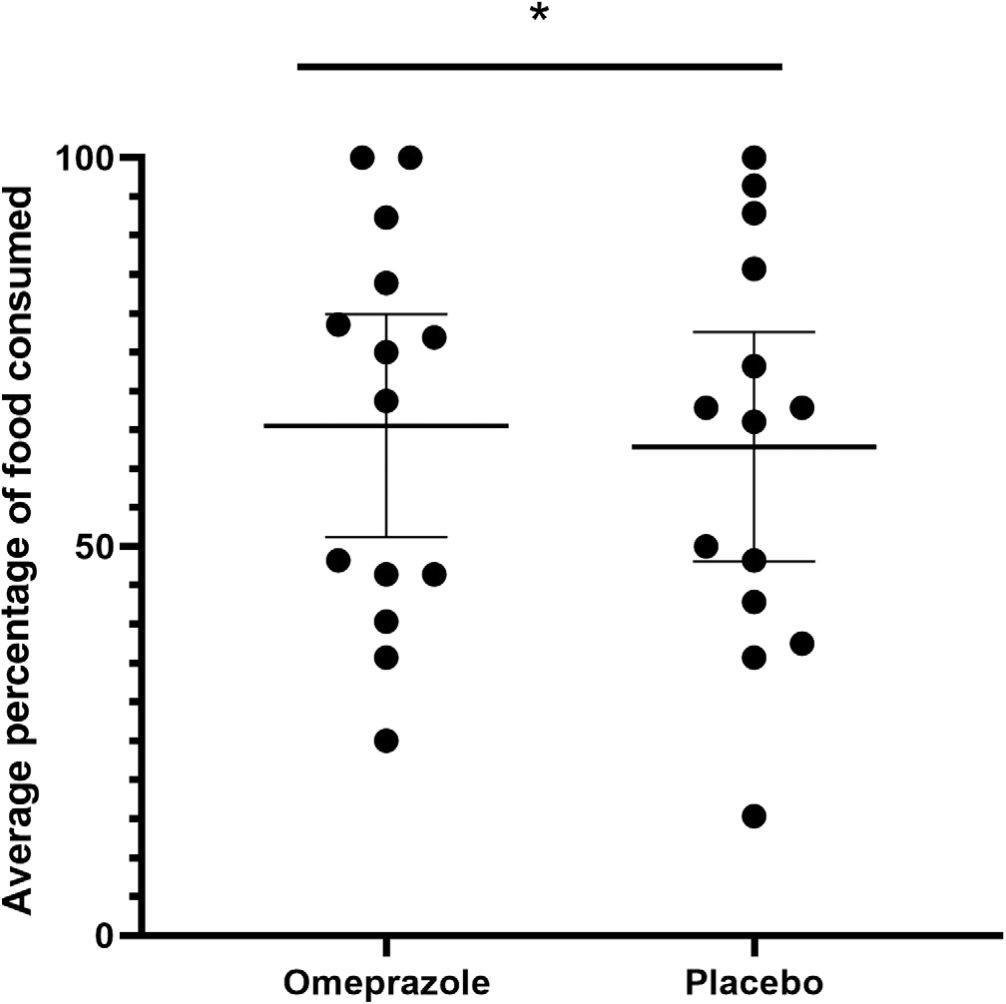
Effect of 2 weeks of omeprazole administration on the average proportion of food offered that was consumed for each in cats with CKD (n = 14) as determined by subjective food assessment. Mean percentage of food consumed and 95% confidence intervals are represented by the horizontal bars for each treatment period. In comparison to cats receiving placebo, there was a small, but statistically significant increase (2.7%) in the percentage of food consumed by the cats while administered at 1 mg/kg of omeprazole PO q24h for 2 weeks (*P = .04). CKD, chronic kidney disease

**FIGURE 3:**
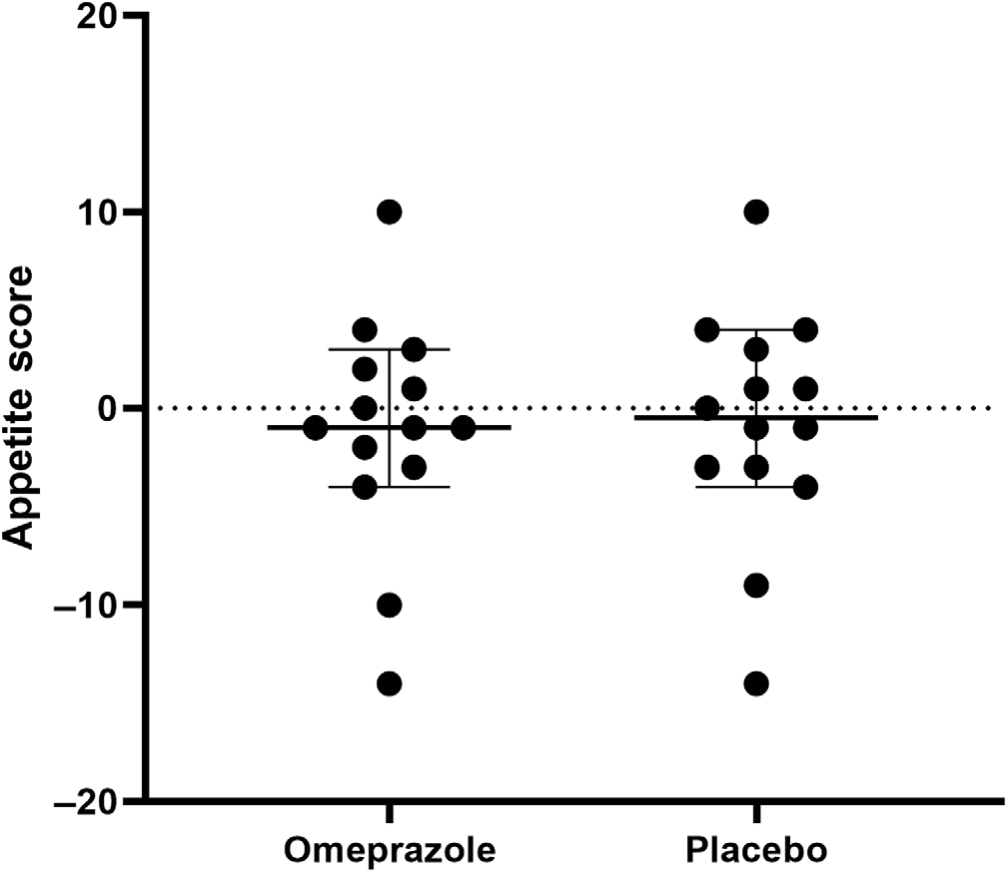
Effect of 2 weeks of omeprazole administration on appetite in cats with CKD (n = 14). Subjective appetite score (sum) and 95% confidence intervals are represented by the horizontal bars for each treatment period. No statistically significant difference in subjective appetite score was observed in cats with CKD PO administered placebo or omeprazole at 1 mg/kg q24h for 2 weeks (*P* = .76). CKD, chronic kidney disease

**FIGURE 4:**
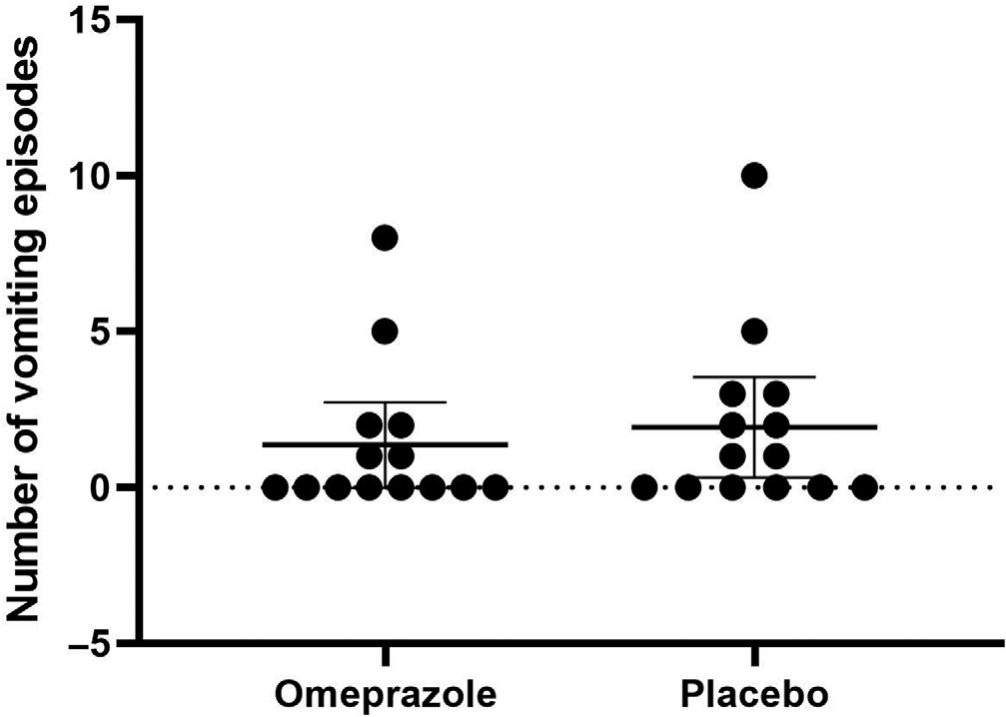
Effect of 2 weeks of omeprazole administration on vomiting frequency in cats with CKD (n = 14). Mean and 95% confidence intervals sum of vomiting episodes for each treatment period are represented by the horizontal bars. No statistically significant difference in frequency of vomiting was observed in cats with CKD PO administered placebo or omeprazole at 1 mg/kg q24h for 2 weeks (*P* = .14). CKD, chronic kidney disease

A significant difference in the subjective appetite score was identified, as assessed by the percentage of food offered that was consumed, between treatments (*P* = .04, Figure 2). The mean food consumed during the 2 weeks of omeprazole treatment (65.55%; SD, 24.87%) was significantly higher than during placebo administration (62.83%; SD, 25.59%). However, no difference was found in the qualitative subjective appetite score (ie, owner perception of the cat's appetite) between treatments (*P* = .76; Figure 3). No difference was observed in either the odds of vomiting or total number of vomiting episodes in CKD cats when given omeprazole in comparison to placebo (*P* = .2 and *P* = .14, respectively; Figure 4). No difference was observed in activity score (*P* = .93), food‐seeking behavior (*P* = .16), or rate of food consumption (*P* = .35).

A small but statistically significant increase in BUN was observed between treatments (*P* = .03). The mean BUN for cats treated with omeprazole was 60.2 ± 18.4 mg/dL whereas mean BUN was 57.2 ± 18.6 mg/dL when treated with placebo. This change was not considered clinically relevant. As expected, serum creatinine concentrations differed between IRIS stages (*P* ≤ .001). Statistically significant differences were not observed in other relevant serum biochemical tests related to renal function or weight after either treatment (Table 1). No differences were observed between IRIS stages for any other measures.

**TABLE 1:** Pre‐treatment and post‐treatment comparison of body weight and relevant serum biochemistry parameters, displayed as mean (SD). No significant differences were noted when comparing pre‐ and post‐treatment body weight, creatinine, BUN, potassium or phosphorus (P > 0.05).

	Pre‐omeprazole	Post‐omeprazole	Pre‐placebo	Post‐placebo
Body weight (mg/dL)	4.6 (2.1)	4.6 (2.0)	4.6 (2.0)	4.7 (2.1)
Creatinine (mg/dL)	3.0 (0.9}	3.4 (1.2)	3.2 (0.9)	3.2 (1.1)
BUN (mg/dL)	57 (16)	60 (21)	57 (18)	53 (20)
K+ (mmol/L)	4.1 (0.4)	3.9 (0.3)	4.1 (0.5)	4.1 (0.4)
Phosphorus (mmol/L)	4.6 (1.6)	5.2 (2.4)	4.8 (2.1)	5.3 (2.3)

No significant differences were noted when comparing pre‐ and post‐treatment body weight, creatinine, potassium, or phosphorus (*P* > .05). BUN differences were observed between treatments (*P* = .03).

Abbreviation: BUN, blood urea nitrogen.

No observable placebo effect was identified when comparing activity and appetite scores, food seeking behavior, percentage of food consumed, or rate of food consumption during placebo administration to following baseline (“rest”) period.

## 4 DISCUSSION

Gastric hyperacidity and ulceration are not commonly identified in cats with CKD.^13^, ^14^ Thus, anecdotal reports from veterinarians and owners of improvement in appetite in cats with CKD are unlikely to be secondary to omeprazole's effect on gastric acid secretion. In vitro and experimental in vivo studies have demonstrated that PPIs, such as omeprazole, have gastric pH‐independent effects, including anti‐inflammatory, antifibrotic, antioxidant, and antineuropathic effects.^15^, ^16^, ^17^ Thus, we chose to evaluate the effect of once‐daily omeprazole administration on subjective improvement of appetite and decreased vomiting in cats with CKD.

In our study, treatment with PO once‐daily omeprazole at 1 mg/kg for a 2‐week treatment period was associated with a statistically significant, but clinically negligible, difference in the average percentage of the offered food consumed as recorded by a subjective assessment. When cats were treated with omeprazole, they had 2.7% higher food intake than when treated with placebo. However, this increase was not detectable by the owners by subjective assessments of appetite, begging behavior, or rate of food ingestion. Furthermore, no change in body weight was detected during the omeprazole administration period although a change in body weight might be hard to detect with such a small increase in percentage of food consumed during the 14‐day omeprazole treatment period.

In several clinical trials in which the same daily log data were collected over a 21‐day period, mirtazapine administration resulted in statistically significant and clinically relevant improvements in subjective appetite scores, body weight, and frequency of vomiting in cats with CKD whereas maropitant only resulted in a significantly decreased frequency of vomiting.^18^, ^19^, ^20^ Cats with moderate to advanced CKD often receive several medications to palliate clinical signs. Given concern for the effects of polypharmacy, which also increases the likelihood of drug interactions, risk for adverse events, and poor owner compliance because of the challenge of medicating cats, other medications shown to be more effective in alleviating poor appetite or vomiting in cats with CKD such as mirtazapine and maropitant should be used as first‐line treatments. Given the small but statistically significant difference in food consumption noted in our clinical trial by use of subjective assessments, omeprazole may be considered as a tertiary addition in the event other antiemetics and appetite stimulants remain ineffective in cats with moderate CKD. In such patients, a clear monitoring period with defined goals should be established to determine if omeprazole treatment is providing a benefit. Additional studies are needed to determine if omeprazole treatment would be more effective in stimulating appetite in cats with IRIS stage 4 CKD, because such patients were not assessed in our study. Moreover, a longer duration of monitoring is recommended because previous studies evaluating the effect of drug treatment on appetite stimulation have used a 21‐day period of monitoring.

Omeprazole is a more potent acid suppressant than histamine‐2 receptor antagonists (eg, famotidine and ranitidine) and has been recommended widely for the treatment of ulcerative gastric or duodenal disease in cats. When treating esophagitis and gastroduodenal ulceration, a dosage of 1 mg/kg PO q12h is recommended to achieve the degree of acid suppression needed to promote mucosal healing.^21^, ^22^ Gastric hyperacidity is not an expected sequela of CKD in cats and pH‐independent effects of PPIs have been observed with once‐daily dosing in rodent models.^15^, ^16^, ^17^ This rationale, combined with the desire to administer the drug at a frequency that would promote compliance, motivated our decision to explore the effect of once‐daily dosing for our clinical trial. The dose of omeprazole used was well tolerated in this population of CKD cats, and no adverse effects were noted throughout the clinical trial. Although we believe it to be unlikely, we cannot discount the possibility that twice‐daily omeprazole dosing would have had a more substantial orexigenic effect.

Overall owner compliance and retention in our study were excellent, with a 96% adherence rate to medication administration. All daily logs were completed, and no patient dropouts occurred because of either poor owner compliance or worsening of clinical disease. The majority of cats received all medications during both placebo and omeprazole treatment periods with 8 cats having had 100% of all treatments administered, 4 having only missed 1 dose of either placebo or omeprazole, and 2 cats having 70% and 89% of total doses administered with 90% of all missed doses in these 2 cats occurring during the placebo treatment period.

This overall high adherence rate was suspected to be related to owner and cat selection at study entry (eg, inclusion criteria included assurance that only 1 owner would administer the drug to the cat and complete the daily log). Clear study guidelines and a lack of observed adverse medication effects were also likely contributors to the high compliance rate. The cause for the difference in missed doses between the placebo and omeprazole treatment periods was unknown. However, it was considered coincidental because the treatment was blinded to the owner and there are no known reasons why the placebo would be more difficult to deliver than the omeprazole treatment.

We recognize the limitation of the small sample of cats enrolled in our study. Our rigorous enrollment criteria for participants resulted in a small number of cats meeting our inclusion criteria. During the 2.5 years of study enrollment, across multiple academic and private practice facilities, several factors led to the exclusion of patients. Common causes of exclusion were instability of kidney disease, instability of chronic or acute concurrent diseases, evidence of comorbidities that could affect the reporting of appetite or vomiting (eg, GI disease, diabetes mellitus), inability to administer medication in the form of a capsule, and owner unwillingness to participate because of the complexity of study design, which required multiple clinic visits. Three study cats previously were receiving once‐daily famotidine; but the owners agreed to discontinue this medication 2 weeks before enrollment. One cat was excluded during the study because the owner felt the drug administered was improving the cat's appetite and therefore decided to drop out of the study and give the cat omeprazole. After unblinding, we determined the cat was receiving placebo. Owners were not asked to weigh food before and after feeding to quantify food consumption. We acknowledge that the study was weakened by not weighing the food before and after feeding and that changes in food intake could have been assessed more objectively and precisely as has been demonstrated previously.^23^ We also used a 5‐point owner‐reported scoring system to assess food consumption in our study, which likely does not provide the same ability to detect small changes in food intake compared to a visual analogue scale using 100‐mm horizontal lines with extremes listed at either end.

No difference was observed in vomiting frequency during omeprazole administration compared to placebo. However, our study was underpowered to identify the true effects of omeprazole on vomiting because only 5 cats were reported to have vomiting at study entry and only 8 cats had ≥1 episode of vomiting during the study period. Future studies to identify the effects of omeprazole on the frequency of vomiting in CKD cats could focus on specifically enrolling patients with vomiting as the primary clinical sign.

Diet was not controlled in our study given the difficulty it would have caused for participants. Although owners were instructed not to change the diet for the duration of the study, cats that routinely ate a variety of foods were allowed to continue to eat that same variety as long as no new foods were introduced. Dysrexic cats with CKD often are fed multiple renal diets, often mixed with nonrenal diets, in an attempt to stimulate appetite. A strict regimen of 1 of these diets could have created a higher risk of worsening inappetence, increased owner stress, and ultimately decreased patient enrollment. The majority of cats included in our study, however, were receiving a therapeutic renal diet. A potential limitation of this approach was its undetected impact on the development of food aversion. Finally, lactose was used to fill the placebo capsules to assist in masking, which is a common practice in drug therapy clinical trials.^18^, ^20^, ^22^ Because some adult cats may be less tolerant of lactose, it is unknown if this factor could have had a negative clinical effect. Although not directly assessed, a negative effect on the patient has not been appreciated in previous studies or clinical trials.^18^, ^20^, ^22^

In our double‐blinded, placebo‐controlled, crossover clinical trial, we showed that once‐daily PO omeprazole at 1 mg/kg had a significant but negligible effect on food consumption when evaluated by a subjective owner assessment score over a 2‐week period in cats with CKD. This difference was so small that it was not appreciated by the owners of the cats. Furthermore, there was no effect on weight gain or activity level. Given the commercial availability of other, more efficacious appetite stimulants, we suggest that once‐daily omeprazole should not be used routinely for the management of hyporexia in cats with CKD in the absence of other comorbidities or concern for GI ulceration.

## CONFLICT OF INTEREST DECLARATION

Authors declare no conflict of interest.

## OFF‐LABEL ANTIMICROBIAL DECLARATION

Authors declare no off‐label use of antimicrobials.

## INSTITUTIONAL ANIMAL CARE AND USE COMMITTEE (IACUC) OR OTHER APPROVAL DECLARATION

This study was approved by the IACUC at North Carolina State University, University of Tennessee, and the Ohio State University (protocol approved 2529‐0517 [UTK]; 19‐037‐O [NCSU]; and 2017A00000073 [OSU]).

## HUMAN ETHICS APPROVAL DECLARATION

Authors declare human ethics approval was not needed for this study.

## Supporting information


Figure S1: Daily log completed by the owners during both rest and treatment periods.
