# Supplementary material for: Appetite‐stimulating effects of once‐daily omeprazole in cats with chronic kidney disease: Double‐blind, placebo‐controlled, randomized, crossover trial
Source: J Vet Intern Med. 2021 Sep 30;35(6):2705–12. doi: 10.1111/jvim.16268 (PMC8692181; doi:10.1111/jvim.16268)
Supplement: Supplementary file 1 — Figure S1: Daily log completed by the owners during both rest and treatment periods. [file JVIM-35-2705-s001.pdf]

Daily Log Sheet

Cat's Name:_________________

Date: ______________________

Did your cat tolerate receiving the medication?

Yes No

Describe your cat's appetite today:

Decreased Unchanged Increased

Of food offered today, my cat ate (circle one):

100% 75% 50% 25% None

How quickly did your cat eat his/her food today?

Slower than normal Unchanged Faster than normal

Describe your cat's activity level today:

Decreased Unchanged Increased

Describe your cat's begging/food seeking behavior today:

Decreased Unchanged Increased

Did your cat vomit today?

Yes No

If yes, please list the number of vomiting episodes in the space provided:

____
